# Toxin-Induced Nodules: A Clinically Distinct Complication With Implications for Aesthetic Practice

**DOI:** 10.1155/crdm/9921924

**Published:** 2025-10-15

**Authors:** Desiree Castelanich, Luis Alberto Parra, Juan Sebastian Rodriguez Cabrales, Eugenia Cure, Eliana Garces, Andrea Marcela Parra

**Affiliations:** ^1^Specialty of Dermatology, Argentine Society of Dermatology, Buenos Aires, Argentina; ^2^Private Practice in Aesthetic Medicine, North University, Barranquilla, Colombia; ^3^Aesthetic Medicine and Clinical Research Practice, National University of Colombia, Bogotá, Colombia; ^4^Aesthetic and Alternative Medicine Private Practice, North University, Barranquilla, Colombia; ^5^Department of Plastic Surgery, Nueva Granada Military University, Bogotá, Colombia; ^6^Department of Ophthalmology-Oculoplastic Surgery, North University, Barranquilla, Colombia

## Abstract

**Background:**

Botulinum toxin type A is widely used for aesthetic purposes and is generally considered safe. However, rare complications such as nodule formation at injection sites can occur, posing diagnostic and therapeutic challenges.

**Aims:**

To describe a case report of patients developing inflammatory nodules after incobotulinum toxin A injections, review the differential diagnoses, and discuss appropriate management strategies based on clinical outcomes and literature.

**Patients/Methods:**

We report three cases of healthy female patients (ages 35–40) who presented with localized nodules approximately 1 cm in diameter following a full-face treatment with Inco botulinum toxin A for treatment of rhytides. Nodules appeared at the frontal injection sites immediately after treatment and were evaluated through clinical examination and ultrasound imaging. Only one patient received empirical anti-inflammatory and antibiotic therapy; the others were managed conservatively with observation alone.

**Discussion:**

All cases resolved spontaneously in one and 2 months, with no scarring or pigmentation changes. Although histopathological confirmation was not performed, the benign clinical course and absence of systemic symptoms suggest a transient inflammatory or hypersensitivity reaction rather than infectious or granulomatous pathology. Literature review supports a multifactorial aetiology, including immune-mediated, foreign-body, and rare infectious causes. Early identification and conservative management are often sufficient, though persistent or atypical cases may warrant biopsy and targeted therapy.

**Conclusions:**

Nodule formation following incobotulinum toxin A injection is an uncommon but important clinical entity. Awareness of its presentation and natural course can prevent unnecessary interventions. Further research is needed to define diagnostic algorithms and management guidelines.

## 1. Introduction

Botulinum toxin injections represent the most frequently performed cosmetic procedure worldwide, as the ISAPS International Survey reported. These treatments are primarily employed for facial wrinkle reduction, rhytide improvement, and management of muscle hypertrophy [1]. Although generally regarded as safe, botulinum toxin administration may rarely result in complications [2]. A large retrospective cohort study demonstrated an overall complication rate of 0.065% for botulinum toxin procedures [3]. The most well-documented adverse effects in upper facial treatments include brow and eyelid ptosis, diplopia, ectropion, lagophthalmos, xerophthalmia, and facial asymmetry [4, 5]. Other documented complications include localized erythema, ecchymosis, oedema, headache, dry mouth, and, rarely, systemic effects such as generalized weakness or dysphagia [3, 4]. Nodule formation represents an underreported nonischemic complication that warrants further attention due to its diagnostic complexity [6].

Among the most important nonischemic complications, we particularly emphasize nodule formation, which typically presents as erythematous subcutaneous lumps that may be either painful or asymptomatic [6]. The pathogenesis of botulinum toxin-associated nodules remains incompletely understood and appears multifactorial, potentially involving infectious agents such as nontuberculous mycobacteria (NTM) [7, 8] or noninfectious processes including foreign-body reactions and sarcoidal granuloma formation [9]. Appropriate management strategies depend on accurate identification of the underlying aetiology, as treatment approaches differ significantly between infectious and noninfectious granulomatous reactions [9]. Despite the global prevalence of botulinum toxin use, consensus guidelines for nodule evaluation and management are absent. This gap is critical, as misdiagnosis may lead to overtreatment (e.g., unwarranted antibiotics) or delayed care for infectious aetiologies.

While nodule formation following botulinum toxin injections is exceptionally rare, recognition of this potential complication carries significant clinical importance. This case report present three patients who developed cutaneous nodules on the forehead following incobotulinum toxin A administration for full-face rhytides in a private dermatology practice setting. This case report contributes to the limited literature on botulinum toxin–associated nodules and provides practical insights for clinicians encountering similar presentations in cosmetic practice.

## 2. Materials and Methods

We present a retrospective case report of 3 female patients between the ages of 35 and 40 who consulted the private cosmetic consultation of Dr. Castelanich in the city of Buenos Aires regarding moderate to severe dynamic rhytides in the face. Medical history was unremarkable, with no contraindications to neuromodulator therapy. The patients had no prior treatments in the upper facial third, with no history of botulinum toxin type A administration or other aesthetic procedures in the forehead region. Informed consent was obtained from all individual participants included in the study for the described treatment and for the publication of their case details and accompanying images.

The patients received a toxin preparation of incobotulinum toxin A (Xeomin®, Merz Pharmaceuticals GmbH, Frankfurt, Germany). For injection, 100 units of Inco botulinum toxin reconstituted in 2 mL normal saline were administered using 1 mL syringes. The needle was inserted to achieve mid-depth placement, with a posterior observation of wheal formation.

All doses and individualized total units per injection point were based on individual patient anatomy, muscle mass, treatment goals, and desired effect, with a mean application of 3 units per injection site.

**Figure 1:**
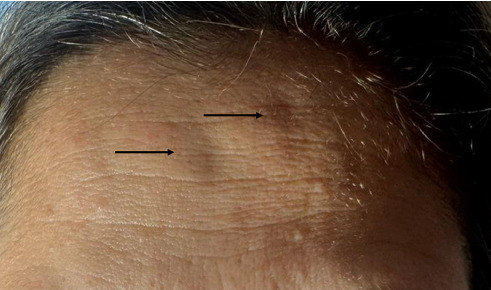
Patient 1: Two nodule formation at incobotulinum toxin A injection sites in the frontal region. Arrows highlight the nodules, measuring approximately 1 cm in diameter, with associated perilesional oedema.

**Figure 2:**
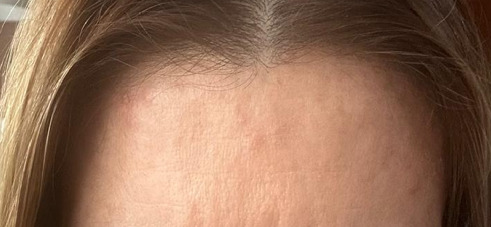
Patient 1: Clinical follow-up demonstrating progressive resolution of toxin-induced nodules. The image shows significant reduction in size of previously documented nodules.

**Figure 3:**
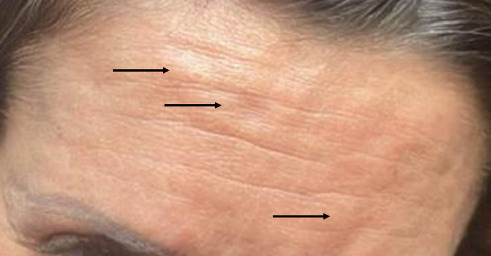
Patient 2: Three nodule formation at incobotulinum toxin A injection sites in the frontal region. Arrows highlight the nodules, measuring approximately 1 cm in diameter, with associated perilesional oedema.

Initially, the treatments were effective, resulting in noticed by the formation of wheals and later the reduction of rhytides. However, immediately after the application of the treatment, the patients observed persistence of inflammation on the forehead. Clinical examination revealed nodules of around 1 cm localized exclusively at the injection sites, with oedema, heat, and swelling associated to both (Figures 1, 2, and 3). Detailed clinical profiles and outcomes for individual cases are synthesized in Table 1.

## 3. Results

**Figure 4:**
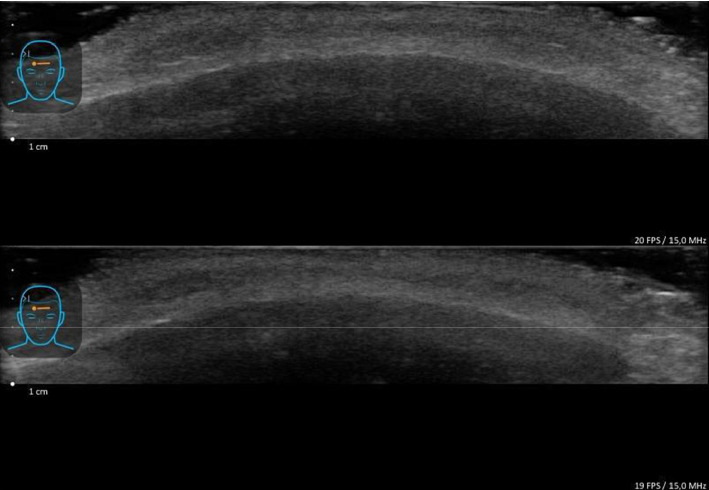
Ultrasound monitoring (15 MHz) of nodule resolution on Patient 1. Both images demonstrate intact anatomical layers with preserved dermal–subdermal junction and normal muscle echotexture, showing absence of fluid collections, granulomatous changes, or other structural complications.

The patients were monitored over a 2-month follow-up period using clinical photography and ultrasound imaging, with no evidence of subcutaneous or deep layer lesions (Figure 4).

Given the small size of the nodules and absence of concerning features (e.g., progressive enlargement), a conservative approach was adopted, with observation recommended over pharmacological or mechanical intervention.

Given the inflammatory presentation of the nodules, we initially considered granulomatous reactions in our differential diagnosis. Patient 1 received empirical therapy with minocycline 100 mg twice daily and loratadine/betamethasone combination (10 mg/0.1 mg daily) for presumed immune-mediated granulomatous inflammation. However, Patients 2 and 3 declined pharmacological intervention, opting instead for close clinical monitoring. Clinical characteristics, management approaches, and resolution timelines for all cases are comprehensively summarized in Table 1.

All three cases demonstrated complete resolution. The patient who received pharmacological treatment resolved around 1 month (Figure 2), while the patients who did not resolved around 2 months with no residual hyperpigmentation or scarring on any of the cases (Figure 3). They initially presented as well-circumscribed 1 cm swellings, which subsequently evolved into asymptomatic subcutaneous nodules.

## 4. Discussion

Botulinum toxin A postinjection nodules have been reported to occur with multiple brands worldwide, reconstituted with commercially available normal saline solution [2]. We present three cases of nodule development in the frontalis after incobotulinum toxin A treatment for full-face rhytides, with self-limited lesions resolving spontaneously within approximately 2 months post-treatment.

Landau et al.'s narrative literature review demonstrates that these nodules exhibit variable onset timing postinjection, consistently correlating with injection sites. While typically appearing within 24 h, emergence may be delayed up to 3 months postprocedure. The lesions generally persist for several days before spontaneous resolution, occasionally recurring in patients with prior uneventful treatments using the same product [10].

The underlying pathogenesis remains unclear, though numerous reports describe botulinum toxin-related allergic reactions manifesting as urticarial papules resembling postinjection nodules, with or without systemic involvement [11–13]. Notably, two cases involving Chinese botulinum toxin A preparations demonstrated positive intradermal tests, with product gelatin content suspected as the allergenic component [14].

Five histologically confirmed granuloma cases following BonTA injections have been documented, including suppurative, sarcoid-like, and foreign-body variants [8, 15–18]. These lesions appeared precisely at injection sites from 2 days to 6 years post-treatment, exhibiting prolonged courses. Experimental forearm injections reproduced sarcoid granulomas with Botox but not saline, suggesting a Kveim reaction mechanism rather than sarcoidosis reactivation [15]. This evidence indicates post-BonTA nodules represent a heterogeneous entity, with most cases demonstrating transient urticaria-like reactions requiring no intervention, while rare instances reflect subclinical granulomatous disease with prolonged duration [10].

These granulomas can be further divided into infectious and noninfectious categories for better treatment purposes. Due to the small number of reported cases, it is difficult to ascertain the exact aetiology of this rare presentation, but several hypotheses have been proposed.

The foreign-body hypothesis proposes that components of reconstituted botulinum toxin solutions may trigger granulomatous reactions. Yun et al. histologically confirmed this mechanism, demonstrating multinucleated giant cells surrounding refractile, nonpolarizable material derived from the proteinaceous solution. While the specific antigenic protein remained unidentified, both xenogenic neurotoxin and human serum albumin were implicated as potential triggers [16].

For suspected infectious aetiologies, we prioritize microbiological confirmation through biopsy with special stains, cultures, and PCR before considering antimicrobial therapy [6, 19]. In cases suggestive of inflammatory pathology, histological confirmation guides appropriate immunomodulatory treatment [19, 20]. However, if the histology shows suppurative or necrotic features, NTM infection should be ruled out using special stains, cultures, and PCR [6]. This systematic approach optimizes outcomes while avoiding unnecessary steroid use in potential infections. For confirmed NTM infections (most commonly *M. abscessus*), we recommend combination antimicrobial therapy including a macrolide like clarithromycin with amikacin, levofloxacin, cefoxitin, or tigecycline for 4–6 months [21–23]. *M. immunogenum* infections have responded successfully to 6 months of clarithromycin monotherapy [24]. While antimicrobial therapy achieved complete remission in five reported cases, three patients developed post-inflammatory hyperpigmentation [25]. Surgical intervention remains reserved for refractory cases despite increased scarring risk [7, 25, 26].

Given frequent false-negative NTM test results, we maintain high clinical suspicion for suppurative granulomas with negative initial workups. In such cases, we advocate repeated sampling or empirical treatment based on strong clinical/histological evidence, as demonstrated by Thanasarnaksorn et al.'s successful 6-month clarithromycin–levofloxacin regimen despite negative initial tests [8, 21, 22].

An alternative immunologic mechanism is proposed by Giavina-Bianchi, who hypothesize that botulinum toxin may stimulate TH1-mediated responses in predisposed individuals. Their case demonstrated both new facial nodules and reactivation of a remote BCG scar, suggesting systemic granulomatous triggering. The authors postulate that this reaction may target unidentified autoantigens, potentially explaining the delayed-onset nodules observed in some patients [19].

Our findings, synthesized with existing literature, support a granuloma-centric pathogenesis model for botulinum toxin nodules: direct foreign-body reactions to protein aggregates or excipients, evidenced by giant cell infiltration of refractile material and systemic immunomodulation triggering latent granulomatous pathways in susceptible individuals.

This dual mechanism carries critical implications: first, it explains the spectrum from immediate hypersensitivity-like reactions to delayed granuloma formation; second, it mandates heightened surveillance for systemic granulomatous activation, particularly in patients with prior BCG vaccination or autoimmune predisposition. The distinct 2-week resolution pattern in our cases suggests transient, localized reactions that may represent a milder variant along this continuum, while persistent nodules likely reflect true granulomatous transformation requiring histologic confirmation.

While this case report provides valuable clinical observations regarding nodule formation after incobotulinum toxin A injections, limitations must be acknowledged. Due to our small sample size, with only three reported cases, the generalizability of our findings is limited. A larger cohort or multicenter study would strengthen the association between incobotulinum toxin A and nodule development.

None of the cases underwent biopsy or immunohistochemical analysis, which could have provided definitive evidence of granulomatous inflammation, foreign-body reaction, or infectious aetiology. However, patients undergoing cosmetic procedures may be reluctant to permit biopsies for nodule evaluation due to concerns about scarring or procedural discomfort, potentially delaying definitive diagnosis. While our cases resolved spontaneously without histopathology, we acknowledge this as a limitation. As emphasized in the literature [2, 10], biopsy is indicated for nodules exhibiting progression beyond 4 weeks, systemic symptoms, inadequate response to conservative management, or suppurative features on ultrasound. In future studies, we recommend protocol-driven biopsies for such cases to differentiate hypersensitivity reactions from subclinical granulomatous disease. This approach would validate our observation that transient nodules represent a distinct entity from true granulomas and provide tissue for microbial PCR.

Due to the quick resolution of nodules, a short follow-up was performed and required no special treatments. However, longer follow-up could reveal late recurrences or persistent subclinical inflammation.

Furthermore, the self-limiting nature of most cases creates a therapeutic paradox: while intervention may be unnecessary, patient expectations for flawless outcomes can drive demands for aggressive treatment of what ultimately proves to be a transient reaction. This underscores the need for noninvasive diagnostic tools and clear communication strategies in cosmetic practice.

## 5. Conclusion

This case report highlights the occurrence of self-limited nodule formation following incobotulinum toxin A injection, a rare but clinically significant complication in aesthetic dermatology. Despite their alarming appearance, all nodules resolved spontaneously within 2 months, suggesting a benign and transient inflammatory response rather than true granulomatous or infectious pathology in these cases.

However, given the growing global use of botulinum toxin products, clinicians should remain vigilant for such presentations and adopt a structured diagnostic approach to distinguish between hypersensitivity reactions, foreign-body granulomas, and infections such as NTM.

Early recognition and appropriate triage are critical to avoid overtreatment or delayed intervention in more severe cases. Our findings underscore the importance of physician awareness, patient education, and judicious use of invasive diagnostic procedures in managing toxin-induced nodules. Further prospective studies and histopathological analyses are necessary to elucidate underlying mechanisms and establish evidence-based guidelines for evaluation and management.

## Figures and Tables

**Table 1:** Summary of clinical features and outcomes in toxin-induced nodules.

Patient	Age	Nodule size/location	Management	Resolution time (months)
1	35	1 cm/frontal	Minocycline 100 mg BID + loratadine/betamethasone	1
2	38	1 cm/frontal	Observation	2
3	40	1 cm/frontal	Observation	2

*Note:* All nodules resolved without scarring or pigmentary changes.

## Data Availability

The data that support the findings of this study are available from the corresponding author upon reasonable request.
